# Infiltration of commercially available, anode supported SOFC’s via inkjet printing

**DOI:** 10.1007/s40243-017-0096-2

**Published:** 2017-05-17

**Authors:** T. B. Mitchell-Williams, R. I. Tomov, S. A. Saadabadi, M. Krauz, P. V. Aravind, B. A. Glowacki, R. V. Kumar

**Affiliations:** 1 Department of Materials Science and Metallurgy, University of Cambridge, Cambridge, United Kingdom; 2 Process and Energy Department, TU Delft, Delft, The Netherlands; 3 Ceramic Department CEREL, Institute of Power Engineering, Boguchwała, Poland; 4 Institute of Power Engineering, 02-981 Warsaw, Poland; 5 Bernal Institute, University of Limerick, Limerick, Ireland

**Keywords:** Inkjet printing, Infiltration, Solid oxide fuel cells, Doped ceria

## Abstract

Commercially available anode supported solid oxide fuel cells (NiO-8YSZ/8YSZ/LSCF- 20 mm in diameter) were anode infiltrated with gadolinium doped ceria (CGO) using a scalable drop-on-demand inkjet printing process. Cells were infiltrated with two different precursor solutions—water based or propionic acid based. The saturation limit of the 0.5 μm thick anode supports sintered at 1400 °C was found to be approximately 1wt%. No significant enhancement in power output was recorded at practical voltage levels. Microstructural characterisation was carried out after electrochemical performance testing using high resolution scanning electron microscopy. This work demonstrates that despite the feasibility of achieving CGO nanoparticle infiltration into thick, commercial SOFC anodes with a simple, low-cost and industrially scalable procedure other loss mechanisms were dominant. Infiltration of model symmetric anode cells with the propionic acid based ink demonstrated that significant reductions in polarisation resistance were possible.

## Introduction

Solid oxide fuel cells (SOFCs) have been the subject of significant research efforts due to their highly efficient conversion of chemical energy into electricity, fuel flexibility and environmental benefits. They have the potential to form a critical part of renewable energy infrastructure both in transportation and at a grid level [1–4].

The state-of-the-art commercial SOFCs are based on a combination of cermet anodes (e.g. Ni-YSZ) and ion-conducting ceramic electrolyte, most often yttria-stabilized zirconia (8YSZ). They operate at temperatures within the region of 800–1000 °C, which allows utilization of the waste heat leading to higher fuel efficiency. Such operating temperatures require use of expensive corrosive resistant interconnects and are detrimental to the durability of the cell due to functional materials degradation. Consequently, the main barriers for the commercialization of SOFCs have been the high cost of production and their operational durability. Thus, the success of SOFC commercialization is critically dependent on the reduction of the operation temperature. However, lowering the operational temperatures leads to a significant drop in SOFC performance due to the increased polarization losses in the electrodes and the decreased ionic conductivity of the electrolyte. The strategies used to compensate for this effect, apart from lowering the electrolyte resistance by using thinner electrolytes, include a reduction of the polarization losses via electrodes nanoengineering [5]. Wet infiltration has been shown to be one of the most effective approaches in enhancing of the electrochemical activity of the anodes and cathodes. Nanodecoration of the electrode surface with particles of a mixed conducting oxide and metal promoters (e.g. gadolinium doped ceria, Ni, Pt, Pd, Cu) has been shown to be a successful method for lowering the operational temperatures and improving the long term stability of SOFCs [6–8]. The concept is based on the infiltration of precursor solutions into the porous electrode scaffold after the high temperature electrolyte sintering step. The formation of the nanoparticles is performed at relatively low temperatures with a simple heat treatment in air. The nanoparticles decoration effectively increases the triple phase boundary (TPB) length and catalyses the electrochemical reactions. Furthermore, the presence of oxide nanoparticles was shown to prevent scaffold particles coarsening and increase the resistance to poisoning from non-pure fuel gases.

Lab scale infiltration of both cathode and anodes has been demonstrated by various groups [9, 10] in laboratory conditions. The infiltration is usually performed with micro pipette drop delivery or sample immersion. The procedure often involves several loading steps with intermediate vacuum treatments to increase the mass load of the infiltrate. An appropriate heat treatment is performed after each step to form either a nanodecoration composed of discrete nanoparticles or interconnected nanoparticle coverage on the electrode scaffold. The process is cumbersome and slow resulting in non-uniform ink distribution (both lateral and in-depth) and waste of expensive ink. Liu et al. [11] infiltrated 1 M water based inks of Sm doped ceria (Sm_0.2_Ce_0.8_O_1.9_—SDC) into laboratory produced anode-supported button cells (NiO-SDC/SDC/Sm_0.5_Sr_0.5_CoO_3_) with an SDC electrolyte densification temperature of 1250 °C. The ink was manually dropped onto the porous anode under vacuum and the infiltration was driven by capillary action. After drying, the pellets were fired at 800 °C for 2 h to decompose the nitrate salts and form corresponding metal oxides. Different loading levels were achieved by repeating the infiltration cycle. It was demonstrated that the cell performances at 600 °C in humidified H_2_ changes with SDC loading. Loadings of 0.96 mmol cm^−3^ (two infiltration cycles) and 1.41 mmol cm^−3^ (three sequential infiltration cycles) led to an increase of the maximum power output by 8 and 22%, respectively. Timurkutluk et al. [12] infiltrated gadolinium doped ceria (CGO) precursor ink into the anode and cathode of NiO-ScSZ/ScSZ/LSF-ScSZ button cells. After five infiltration passes it was found that at 700 °C the cell impregnated with 1.5 M solution provides peak power density of 1.34 W cm^−2^ compared to the cell without impregnation producing only 0.78 W cm^−2^. They also noticed that infiltration with more concentrated precursor solutions reduced power output due to a reduction in open porosity. Sholklapper et al. [13] reported that infiltration of SDC into a composite NiO-ScSZ anode led not only to an increase in fuel electrode performance (from 348 to 403 mW cm^−2^) but more importantly to a significant increase in sulphur tolerance. Single step infiltration of the cathode of commercially available cells has been reported by Dowd et al. [14]. They could load ~8–10 wt% La_0.6_Sr_0.4_CoO_3-d_ (LSCo) into a composite LSCF/SDC cathode that had a thickness of ~50–60 μm in a single step using an ultrasonic spray nozzle. The samples showed improved power performance and increased durability at 200 h operation compared to a reference. Jiang [15] provided a comprehensive review of the benefits and challenges facing infiltrated SOFC electrodes. Based on data from various authors the improved performances of the anodes infiltrated with mixed conducting oxides could be attributed to the extension of TPB as well as enhanced catalytic activities of the anodes.

For the infiltration to be incorporated industrially the process needs to be commercially viable. Inkjet printing is an inherently scalable, low-cost and controllable technique delivering nano- to pico-litre drops with kHz frequency over large areas with micrometre positioning precision. We have shown it to be an effective technique for fabrication and infiltration of SOFCs [16–18]. The efficient use of potentially expensive precursor materials and reproducibility of the inkjet infiltration process are significant advantages for both research and scale-up. Additionally, the non-contact nature allows more delicate electrolyte supported cells to be infiltrated safely whilst the drop impact velocity, in the range or 1–2 m s^−1^ [19] improves infiltration without having to employ vacuum processing.

The current work studies the feasibility of inkjet printing infiltration of anode supported SOFC produced via commercial SOFC technology. The aim of the research was to reduce the number of infiltration processing steps. The goal was to achieve deep penetration of the ink into the thick anode supports avoiding vacuum infiltration treatments and to ensure uniform lateral delivery of the ink with nanolitre precision.

The performance of two inks, one propionic acid based and another water based were investigated. Many research groups report results from water based solution infiltration [15], whilst our previous work showed how propionic acid based inks can be used to form doped ceria [20]. Propionic acid based solutions spread well on metal and oxide surfaces [21] and so, unlike aqueous solutions, do not require additional surfactants to achieve a high degree of wetting. Hence, there was interest in comparing the performance enhancement for alternative precursor solutions, particularly where the rheological properties for each ink may be different. In addition to fuel cell infiltration, symmetric anode cells were produced and infiltrated to demonstrate the suitability of propionic acid ink precursor.

## Experimental

### Solution ink preparation

Two gadolinium doped ceria (CGO) precursor inks were prepared based on different solvents: water (H_2_O) and propionic acid (PPA). Both were produced using cerium nitrate hexahydrate (99.999%, Alfa-Aesar) and gadolinium nitrate hexahydrate (99.9%, Alfa-Aesar). A stoichiometric quantity of the salts (Ce_0.9_, Gd_0.1_) were dissolved in the solvent to produce a stock solution with a total metal ion concentration (TMIC) of 1.5 M. The water based ink had a surfactant (Triton X-100, Sigma Aldrich) added (3wt% of nitrates used) to improve printability and wetting characteristics. The inks were then diluted to lower TMIC concentrations to achieve optimised rheology for inkjet deposition. The ink details and properties are summarised in Table 1.

**Table 1:** Ink details and properties

Primary solvent	Concentration after dilution (M)	Additives	Viscosity (cP)
Water	1.0	Triton© X-100, 3wt % nitrates	1.8
Propionic acid	0.5	None	2.2

Viscosity was measured using a programmable viscometer (LVDV-II+, Brookfield) fitted with a small sample adaptor and spindle (SC4-18, suitable for viscosities 1.5–30,000 cP). Rotation speeds were scanned from 20 to 160 RPM, corresponding to shear rates of 26 and 211 s^−1^, respectively. No shear thickening or thinning behaviour was observed for either ink.

### Jetting

A single inkjet printing nozzle (100 μm diameter ruby orifice, electromagnetically driven Domino Macrojet printhead) was used to produce symmetric anode cells and infiltrate the anode supports of the fuel cells. The pressure applied to the fluid reservoir and the opening time of the nozzle orifice were optimised to dispense well defined drops.

To ensure accurate and precise jetting and to fully characterise the jetting process drop visualisation and optimisation was performed. The system for visualisation consisted of a collinear LED strobe and camera (Stingray F-125B, Allied Vision Technologies) fitted with a zoom lens (ML-Z07545, Moritex). The LED backlit the drops in flight. The nozzle and camera shutter were precisely triggered with increasing delay times between nozzle and camera to image the entire jetting process. The images were then analysed using in-house software that quantified the drop volumes and velocities. This enabled optimisation of the jetting parameters to produce desired drop formation and ensure equivalent quantities of ink were uniformly distributed on the surface of each anode support.

### Contact angles

A micropipette was used to dispense a single 10 μL drop of each ink onto flat, dense, polycrystalline substrates representative of the anode components: yttria-8 mol% zirconia (8YSZ) and nickel oxide (NiO). The drop visualisation system was used to image the sessile drop and the drop spreading after deposition. Contact angles were measured by analysing images using ImageJ [22].

### Full cell preparation

The cells used were commercially available anode supported solid oxide fuel cells (CEREL, Poland). They had a composite nickel oxide/yttria-8 mol% zirconia (NiO/8YSZ) anode, 8YSZ electrolyte and lanthanum strontium cobalt ferrite (LSCF) cathode. The anode thickness was approximately 500 μm with an electrolyte and cathode total thickness <25 μm. The anode diameter was 20 mm and the cathode was 17 mm, giving an effective area of 2.26 cm^2^.

Two samples were infiltrated: one with the water based ink and the other with the PPA based solution. A third cell was left unaltered to act as a reference. The choice of optimum jetting parameters for each ink was also driven by the condition to have similar drop volumes and drop velocities for both inks. Thus, the difference in the performance could be attributed only to the difference in the inks rheological parameters. Inks were deposited on to the anode surface at room temperature using jetting parameters that gave drop volumes of approximately ~19 nL and velocities of ~1.5 m s^−1^. Drops were deposited across the entire anode in a square array pattern with a spacing of 1 mm. For the PPA based ink passes were repeated until the ink was no longer absorbed into the porous anode (4 passes). Two passes were used for the H_2_O ink to reflect the higher metal ion concentration. No vacuum treatment was employed at any stage of the infiltration procedure. Following the first ink deposition step the cells went through a brief thermal treatment to remove organic components in the anodes. The cells were heated to 500 °C in air with heating and cooling rates of 5 °C min^−1^. The room temperature ink deposition was then repeated with the same number of passes. The final stage was a higher temperature calcination, where the cells were heated to 800 °C in air and held for 30 min. Heating and cooling rates were 5 °C min^−1^.

The cells were weighed (SI-234 Analytic Balance, Denver Instrument) before and after the infiltration process to determine the mass loading of CGO. In both cases the infiltrated cells had approximately 1 wt% CGO in the anode following infiltration and calcination.

### Symmetric anode cell preparation

#### Suspension ink

A suspension ink of NiO:CGO (60:40 wt%) was formed by milling 10 mol% gadolinium doped cerium oxide powder (99.9%, Sigma Aldrich) and NiO (99%, Sigma-Aldrich) powders for 8 h. The powders were dispersed in a mixed solvent mixture of methanol and terpineol (50:50 vol%) and ethyl cellulose (99.9%, Sigma Aldrich) was added as a polymeric dispersant.

#### CGO electrolytes

CGO pellets were formed from 10 mol% gadolinium doped cerium oxide powder (99.9%, Sigma Aldrich) milled with 3 wt% hydroxypropyl cellulose (Sigma-Aldrich, average molecular weight: 10,000) for 4 h. Pellets were pressed using 0.350 g of powder in a 12.5 mm diameter die under 3 tonnes before sintering at 900 °C in air. CGO pellets were used due to their higher ionic conductivity relative to 8YSZ at intermediate temperatures, therefore, allowing thicker, more robust symmetric cells to be produced.

#### Symmetric anode deposition

Symmetric button cells were fabricated by depositing the NiO/CGO suspension ink on to the surface of the CGO pellets. Drops were deposited in a square array pattern with 0.6 mm spacing and 0.2 mm offset between printing passes to avoid drop replica stacking. The deposition was performed on a hot stage at 90 °C. Six printing passes were performed on each side to give anodes with thicknesses of ~10 μm. The anodes were sintered in air at 1100 °C.

#### Symmetric anode infiltration

Cells were infiltrated on both sides with the propionic acid based ink. Infiltration was performed using a 1 mm square array pattern across the entire surface with the substrate at room temperature. Following every 2 print cycles an intermediate heat treatment was performed where the sample was heated to 350 °C before cooling back to room temperature. This was to burn out organic residues and increase the loading fraction possible. A maximum of 20 printing passes was performed on each side leading to a maximum mass loading of ~30 wt%.

Following infiltration both the infiltrated and reference samples were heated to 1400 °C in air to simulate the conditions used during typical electrolyte sintering. Following sintering the anode area was 1 cm^2^.

### Characterisation

#### Fuel cell electrochemical characterisation

Electrochemical performance was characterised using an Open Flange test set-up (Fiaxell SOFC Technologies) with polarisation curves measured using a potentiostat (Gamry Instruments) controlled using proprietary software (Gamry Instruments Framework).

Cells were reduced at 800 °C for 2 h then tested in pure humidified hydrogen. The humidity was controlled by bubbling the fuel gas through a water bath held at 30 °C, corresponding to an equilibrium humidity of ~4%. The flow rate was 100 mL(*n*) min^−1^. Nickel and gold mesh acted as the current and voltage taps for the anode and cathode, respectively. Sealing was achieved compressively against alumina felt on the cathode and against mica sheet at the anode. With the size of fuel cell used and the compressive sealing there was a small, consistent, leak during all cell tests.

#### Symmetric cell electrochemical characterisation

Cells were reduced at 800 °C for 2 h prior to measurement. AC impedance measurements were performed using a Solartron impedance analyser system (SI1260 + SI1287) using a two-electrode configuration. The EIS scans from 0.1 Hz to 1 MHz, with an applied voltage of 10 mV and no bias, were conducted at a temperature of 600 °C under 4% humidified hydrogen (balance argon) with a gas flow rate of 30 ml/min. Z view software (Solartron) was used to obtain Nyquist plots. Stability testing was also performed on an infiltrated sample for 50 h at 600 °C.

#### Microstructural characterisation

Microstructural characterisation was carried out after electrochemical performance testing. For full fuel cells using high resolution scanning electron microscopy (FEI Nova NanoSEM). Samples were fractured and mounted with the exposed surface visible to determine the size and distribution of the nanoparticles in the anode. Image analysis for nanoparticle size was performed using ImageJ [22]. Symmetric cells were imaged using a JEOL JSM 6430 SEM in plan view.

## Results and discussion

### Jetting

The drops jetted towards the surface of the anode support had a defined kinetic energy prior to their contact with the porous medium, as discussed in [19]. Hence, while the axial momentum of the impact was transformed to radial spreading, the pressure of the impact facilitated the ink penetration into the substrate. Next, capillary effects have drawn the ink into the porous support. Reis et al. [23] presented a numerical model studying the dynamics of the impact/absorption of a liquid drop on a porous medium. Spreading and penetration of the drop into a porous medium was found to be governed by several parameters amongst which several have practical importance to our experimental scenario—Reynolds number (*Re*), Weber number (*We*), porosity (*ε*) and contact angle (*θ*).

$$Re = \frac{{\rho u_{0} r_{0} }}{\mu };We = \frac{{\rho u_{0}^{2} r_{0} }}{\gamma },$$

where *u*_*o*_ is the drop impingement velocity, *r*_*o*_ the drop radius at impact, *ρ* the specific mass of the ink, *μ* is the viscosity and *γ* the surface tension.

*Re* can be thought of as the ratio of inertial to viscous forces whilst *We* gives a measure of the relative importance of the inertia to the surface tension. The model results of Reis indicated that *Re* is more related to the amount of momentum dissipation, while *We*, *ε* and *θ* can be mainly related to capillary pressure. Consequently, the penetration depth and spreading of the ink in the infiltration procedure performed in our experiments were found to be a complex function of fixed parameters (*ε*), parameters with limited variability (*ρ*, *μ, γ* and *θ*) restricted by the rheological window of stable jetting for the print-head in use and sufficiently variable parameters (*u*_*o*_, *r*_*o*_). Hence, effort was directed towards achieving high impingement velocity (*u*_*o*_) simultaneously preserving stable jetting without the formation of satellite drops or splashing. Smaller drop volumes (*r*_*o*_) were considered desirable to ensure more uniform delivery of the ink over the anode support surface. Additionally, the uniformity of the ink distribution was controlled by the optimum choice of the printer lateral step size and the overlap between the drop replicas on the surface.

**Fig. 1:**
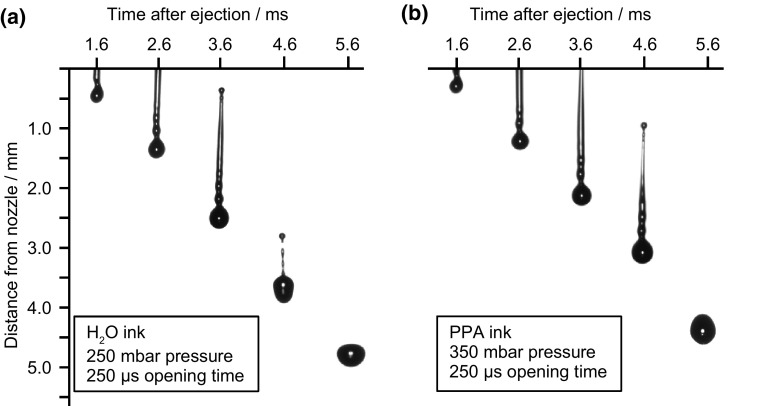
Time sequence illustrating drop formation for the (**a**) water based ink and (**b**) the propionic acid based CGO ink. The images shown are for the printing parameters used during infiltration. The formation of a single drop with a known volume and velocity was achieved by adjusting the ink reservoir pressure and nozzle opening time

**Fig. 2:**
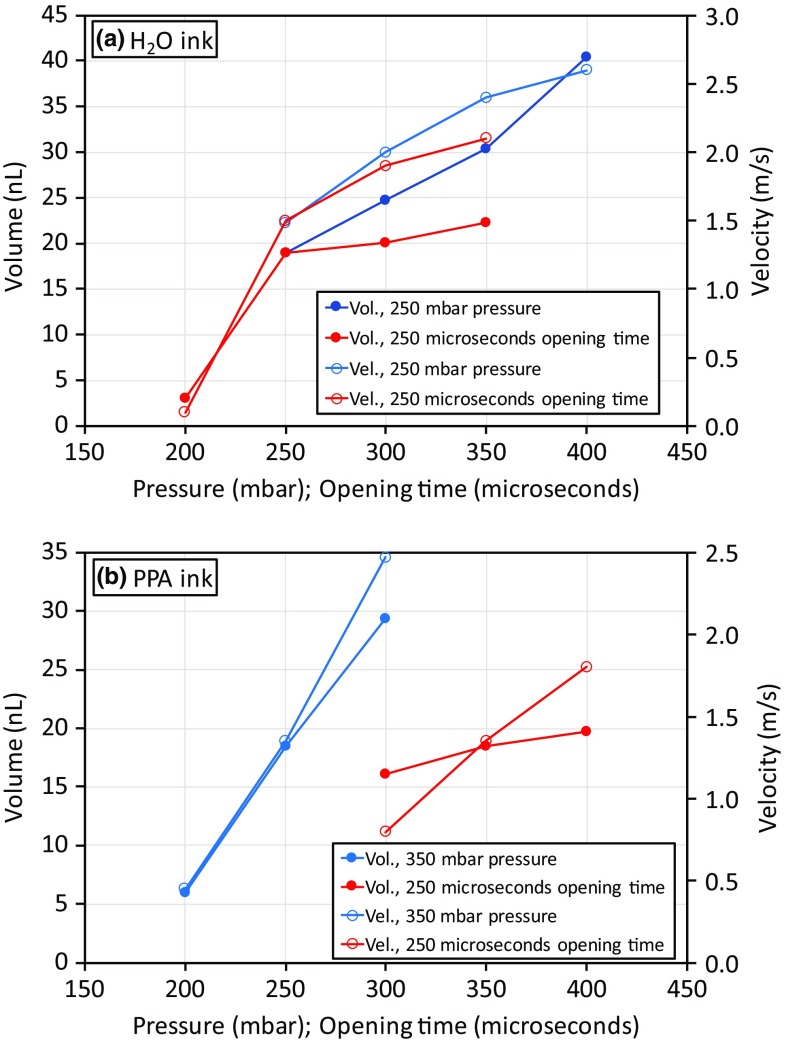
*Plots* illustrating how the adjustable printing parameters affected the drop volume and velocity for (**a**) the water based ink and (**b**) the propionic acid based CGO ink. Series are plotted showing how both drop volume and velocity vary with opening time for a fixed pressure and vice versa. Higher pressures and longer opening times both give higher velocities and larger drop volumes

Drop visualisation enabled both ink jetting parameters to be tailored in such a way that each triggering event resulted in a single drop, without satellite drops, before reaching the substrate. Figure 1 shows images of optimised jetting of the drops in flight for both the water and propionic acid based CGO inks. For both inks the initial drop forms an elongated tail after it detaches from nozzle, but the tail part catches up to form a single drop. Hence at optimised jetting parameters no satellite drop formation is achieved. The optimal parameters for the water based ink were 250 mbar pressure and 250 µs opening time and 350 mbar pressure and 250 µs for the propionic acid ink. Figure 2 shows how the parameters of applied ink reservoir pressure and nozzle opening time influence the drop volume and drop velocities. Higher pressures and longer opening times both lead to larger drop volumes and greater velocities. The extreme values for both volume and velocity are constrained by the ability to reproducibly deposit well-formed droplets. When very low pressures or opening times were used, jetting is inconsistent and drop velocities were very low leading to poor accuracy at the substrate. When very large values for opening time and pressure were used, very long ligaments formed and broke up into many satellite drops resulting in poor control of placement and volume.

### Contact angles

**Fig. 3:**
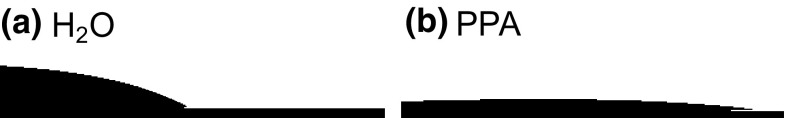
Photographs illustrating the difference in contact angle on polycrystalline, dense 8YSZ between (**a**) the water based ink and (**b**) the propionic acid based ink. The PPA ink showed better wetting and a lower static contact angle

**Fig. 4:**
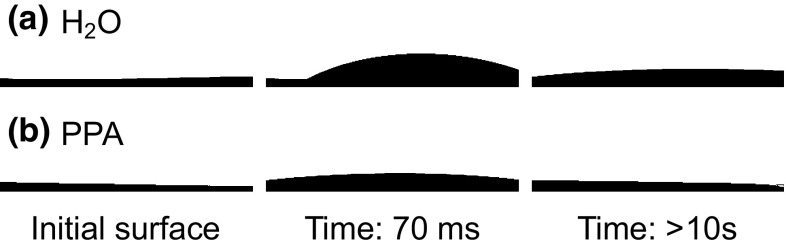
Time-series of photographs illustrating the wetting behaviour on NiO for (**a**) the water based and (**b**) the propionic acid based ink. Whilst both inks wet the surface well the PPA based ink spread further

The contact angle on relevant substrates was found to be lower for the propionic acid ink than the water based ink, Fig. 3. The static contact angle was 24° for the water based CGO ink and 6° for the propionic acid based ink on 8YSZ. Both inks wetted the NiO surface better than the 8YSZ such that it was not possible to measure a static contact angle on the NiO substrate. In Fig. 4 a time-series of photographs illustrates how drops of both CGO inks wet the NiO surface. These results indicate that the PPA ink is likely to have spread over the surface of the anode more uniformly and, therefore, should lead to a better distribution of the CGO nanoparticles.

### Full cell microstructure

**Fig. 5:**
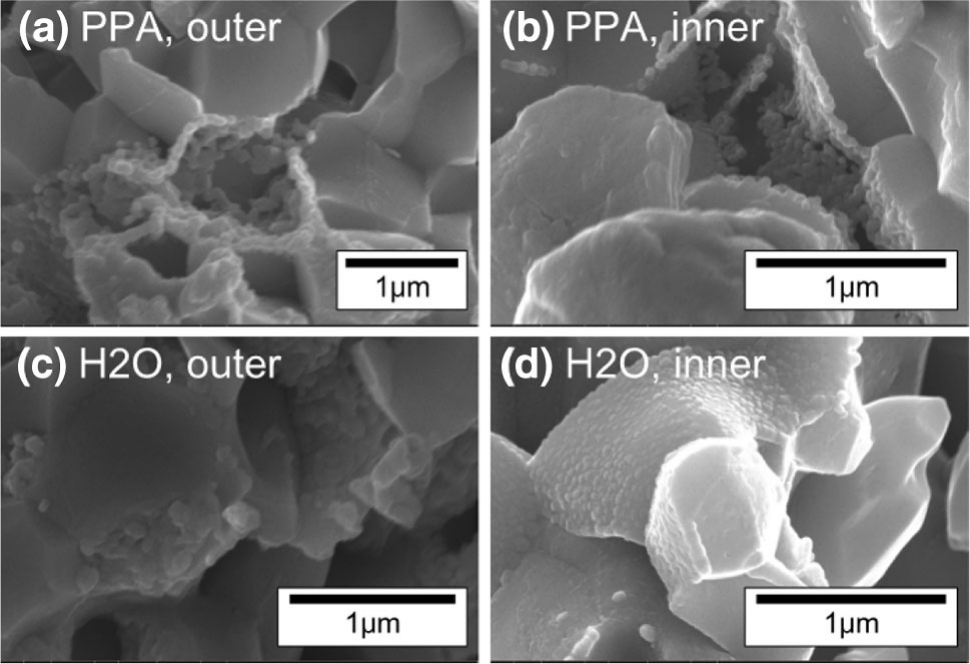
Secondary electron images showing the fractured cross-section of the fuel cell anodes. CGO nanoparticles decorate the anode surface for (**a**, **b**) the PPA and (**c**, **d**) the H_2_O infiltrated cell. Both at the outer surface, (**a**, **c**) and near the electrolyte interface, (**b**, **d**)

The presence and distribution of CGO nanoparticles was studied by high resolution SEM. It can be seen in Fig. 5 that the CGO nanoparticles had a similar size and shape in both infiltrated samples. The size of the nanoparticles varied from approximately 40 nm to 100 nm. An average of 120 particles gave a mean of 56 nm and standard deviation 16 nm.

Both also showed an approximately constant distribution of nanoparticles throughout the thickness of the anode from the outer surface to the electrolyte interface. The formation of CGO nanoparticles near the anode/electrolyte interface was the main target of the infiltration experiment as previous modelling studies have shown that the electrochemical reaction processes occur within ~20 μm of the interface [24].

### Full cell electrochemical performance

**Fig. 6:**
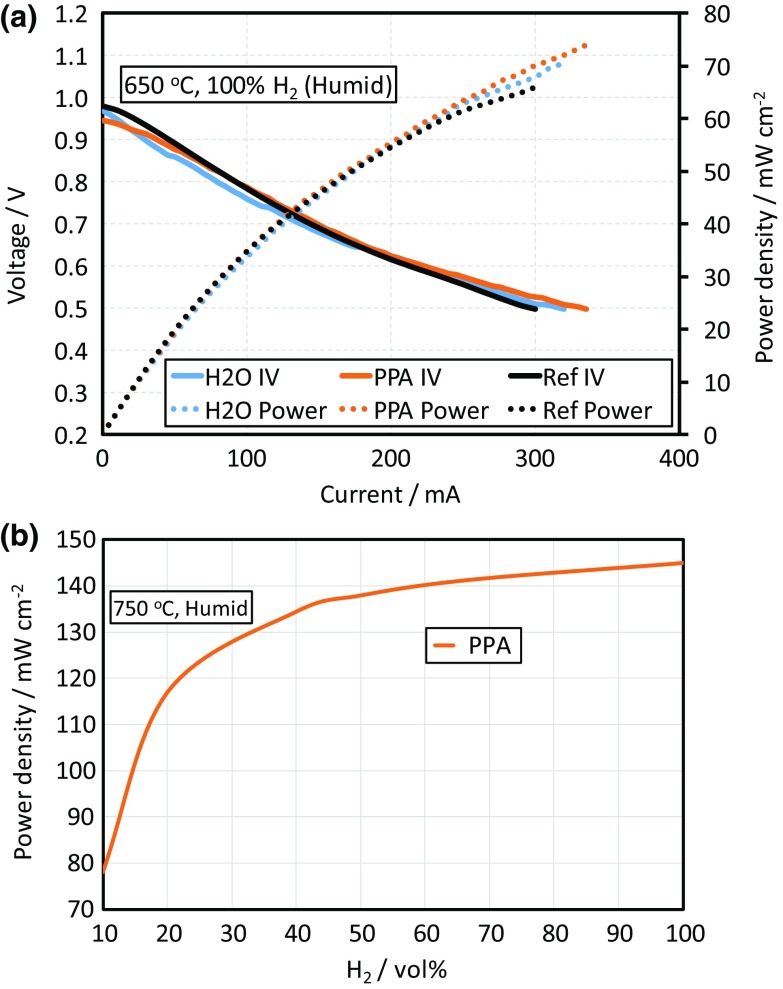
**a** I-V characteristics and resulting power curve for both infiltrated and reference fuel cells. The infiltrated cells only show a small improvement in the maximum power output relative to the reference cell at impractically low voltages. **b** The power density shows a sharp decrease when the fuel concentration is reduced indicating high concentration losses due to low open porosity in the cells

The polarisation curve and power density for each of the cells is shown in Fig. 6a. The samples infiltrated with the CGO precursor solution show a small improvement in the maximum power measured. The power density was approximately 7 and 12% higher for the H_2_O and PPA samples, respectively, relative to the reference cell, but only at low voltages. The measured open circuit voltages (OCV) were lower than the theoretical expected value and indicated a small, but consistent, leak in the testing system. However, despite the leak all three samples show approximately the same OCV (950–980 mV). With the best performing cell, the PPA infiltrated sample, showing the lowest.

The results suggested that the presence of 1 wt% CGO in the anode only affected the power output of commercially available fuel cells at low cell voltages. The power output, for the PPA infiltrated cell, at 750 °C measured at different H_2_/N_2_ volume ratios is presented in Fig. 6b. The sharp rather than gradual decline in the power density with the reduction of H_2_ gas vol% suggested the presence of substantial concentration losses, most likely due to the low porosity of the anode support.

The lack of substantial performance improvement for the infiltrated samples at suitable cell voltages was attributed to two factors: the relatively low nanoparticle loading and the relatively small contribution to total loss from the anode polarisation resistance. In symmetric cells when a large CGO particle loading fraction is achieved the polarisation resistance of the anode is decreased significantly. However, in the real fuel cells there will also be loss contributions from the cathode polarisation resistance and concentration losses. Therefore, the small reduction in anode polarisation resistance may be dwarfed by the other loss mechanisms in this case.

The small difference between the performance of the H_2_O and PPA infiltrated samples at higher current densities can be in part explained by the connectivity of the CGO nanoparticles. Although the particles are of similar sizes and shapes, the CGO in the PPA sample shows greater connectivity than in the H_2_O, Fig. 5b and d, providing a better percolative path through the anode scaffold. The small difference in the CGO particle morphology was ascribed to the higher surface tension of water compared to propionic acid, which was reflected by the contact angle measurements presented in Sect. “Contact angles”. This may have led to more isolated precursor residue replicas during the drying process and, therefore, less connected CGO nanoparticles.

To improve the performance more significantly the cathode should also be infiltrated to reduce the polarisation resistance of both electrodes. Kiebach et al. [25] infiltrated cells assembled into a stack by flooding the electrodes using the gas manifolds to deliver the aqueous CGO and CGO/Ni precursor solutions. They found an improvement in stack performance following cathode infiltration but no significant further improvement after subsequent anode infiltration. In fact, the flooding method led to poor electrical contact with one of the cells due to accumulation of CGO at the bottom of the stack and, therefore, a reduction in the total power output. The precise deposition achievable with an inkjet printing approach reported here avoids those inherent materials wastage and accumulation problems. However, our results broadly agree; anode infiltration alone did not significantly enhance the electrochemical performance of commercial cells at intermediate temperatures, due to the dominance of cathode losses [26]. The benefit of anode infiltration in commercial cells may be more pronounced for long term stability and fuel impurity tolerance [8, 27, 28].

Furthermore, using a more porous anode structure would benefit the cell in two ways: it would reduce the concentration losses occurring due to limited gas diffusion and increase the fraction of nanoparticle loading achievable.

To determine the effectiveness of the infiltration procedure on enhancing the anode performance, symmetric anode cells were produced and tested. This enabled the anode losses to be separated from the more dominant cathode and concentration losses. Therefore, verifying that the propionic acid ink precursor route was suitable for solution infiltration.

A CGO electrolyte was used due to the higher ionic conductivity at intermediate temperatures [29]. This meant a thicker electrolyte could be used, which facilitated easy production and handling. A composite NiO/CGO anode was used to ensure a compatible thermal expansion coefficient with the electrolyte. Thin anodes, ~10 μm, prevented significant concentration losses.

### Symmetric cell results

#### Symmetric cell microstructure

**Fig. 7:**
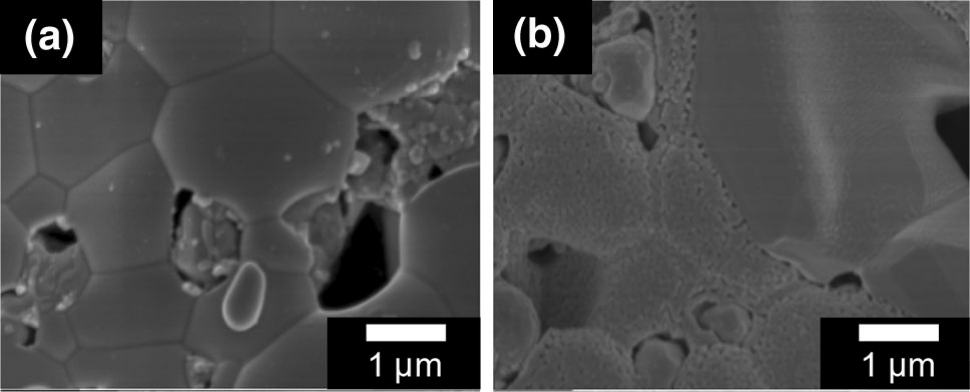
Secondary electron image showing the symmetric anode structure in plan view (**a**) before and (**b**) after 8 infiltration printing passes. The infiltrated sample had a high density of CGO nanoparticles decorating the surface

Following infiltration, the symmetric anodes were found to have a high density of CGO nanoparticles decorating the surface, Fig. 7, even after 8 printing cycles. This indicated that the PPA ink could effectively deliver precursors for forming well distributed nanoparticles.

#### Symmetric cell impedance

**Fig. 8:**
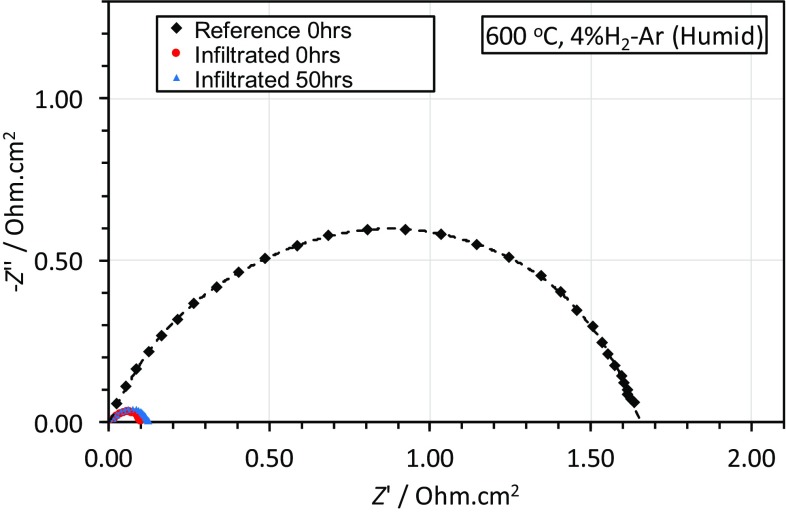
Nyquist plots for symmetric anode cells tested at 600 °C. The size of the arc indicates the magnitude of the polarisation losses. The infiltrated cell showed much lower polarisation losses than the reference both before and after 50 h aging

The symmetric cell infiltrated with CGO using 20 printing cycles of the propionic acid ink demonstrated a lower polarisation resistance, Fig. 8, than the reference cell. The smaller polarisation resistance arc for the infiltrated sample indicates that the infiltrated CGO nanoparticles effectively extend the triple phase boundary (TPD) and, therefore, geometrically enhance the electrode performance.

The Nyquist plots were shifted to subtract the ohmic component, which depends on electrode contact and electrolyte thickness, for clarity. The area specific resistance (ASR) values were estimated using the real axis intercepts and are summarised in Table 2. The infiltrated cell had an ASR that was more than 10 times lower than the reference cell before and after aging. The lack of significant performance degradation after 50 h was promising and demonstrated that at 600 °C the nanostructured anode was stable.

**Table 2:** Area specific resistances of the symmetric cells

Sample	Area specific resistance (ASR) (mOhm cm^2^)
Reference	830
Infiltrated: 0 h	50
Infiltrated: 50 h	60

## Summary

The current work reports a two-step, scalable, inkjet printing infiltration process to infiltrate CGO nanoparticles into commercial SOFC anodes. Two inks, one water and one propionic acid based, were deposited using a well-controlled inkjet printing process. The propionic acid ink was found to wet the surface of relevant anode materials more extensively.

No significant difference in power output was measured at practically relevant voltages. This was ascribed to other loss mechanisms including cathode polarisation and concentration losses being more dominant.

Symmetric anode cells were also fabricated and infiltrated with the propionic acid based ink. These cells demonstrated that the propionic acid ink was an effective precursor for CGO nanoparticle formation and that when anode polarisation losses were isolated the infiltrated cells performed significantly better than a reference cell. The infiltrated cell also showed no significant degradation after aging for 50 h at 600 °C, with the polarisation resistance remaining an order of magnitude lower than the uninfiltrated cell.

Future work will look to increase the mass loading of CGO and incorporate both cathode and anode infiltration. More porous anodes should enable greater loading and reduced concentration losses. Additionally, the effect of infiltration on long-term stability will be reported.
